# The effect of bariatric surgery on dietary Behaviour, dietary recommendation Adherence, and micronutrient deficiencies one year after surgery

**DOI:** 10.1016/j.pmedr.2023.102343

**Published:** 2023-07-22

**Authors:** Alaa H. Qadhi, Asma H. Almuqati, Nuha S. Alamro, Afnan S. Azhri, Firas S. Azzeh, Wedad F. Azhar, Reema A. Alyamani, Najlaa H. Almohmadi, Sarah O. Alkholy, Walaa E. Alhassani, Wafaa F. Abusudah, Abrar M. Babateen, Bayan Tashkandi, Nouf A. Alharbi, Abed H. Al-Slaihat, Khloud J. Ghafouri

**Affiliations:** a Department of Clinical Nutrition, Faculty of Applied Medical Sciences, Umm Al-Qura University, Makkah, Saudi Arabia; b Clinical Nutrition Administration, King Abdullah Medical City Hospital, Makkah, Saudi Arabia; c Food and Nutrition Department, King Abdulaziz University, Jeddah, Saudi Arabia; d Department of Nutrition and Food Science, Northern Border University, Arar 91431, Saudi Arabia; e Department of Nutrition and Food Technology, School of Agriculture, The University of Jordan, Amman, Jordan

**Keywords:** Bariatric surgery, Eating behaviour, Emotional Eating, Makkah

## Abstract

Bariatric surgery (BS) is associated with vitamin and mineral deficiencies, which might be augmented by low adherence to dietary guidelines and inappropriate dietary behaviours. The aim of this study was to determine the influence of BS on eating behaviour, prevalence of nutrient deficiency, level of commitment to diet, and lifestyle recommendations one-year post-BS.

A cross-sectional study was conducted among adult patients who underwent BS in 2019 and had follow-up for a year. Age, gender, and clinical data were collected from the hospital system and other information was obtained from questionnaires during phone interviews. A total of 160 patients participated in the study.

At 12 months, a significant increase from the baseline values in plasma levels of vitamin B_12_, folate, vitamin D, iron, corrected calcium, albumin, CRP, and MCV, as well as a significant decrease in BMI was observed. Adherence to dietary and lifestyle recommendations was moderate to high. Emotional, and restrained eating behaviours were moderate with 64.4%, and 77.5%, respectively. External eating was low at 58.1%.

The study concluded that pre-and post-bariatric surgery nutrients should be closely monitored.

## Highlights

• Bariatric surgery is associated with nutrient deficiencies besides weight reduction.
• A good level of adherence to dietary guidelines was observed for bariatric patients.
• Patients showed a firm commitment to avoiding high-calorie diets.
• About two-thirds of the participants had a moderate level of emotional eating.
• Around half of the participants had a low level of external eating.

## 1 Introduction

Obesity has become a major health concern in Saudi Arabia. (Alkhaldy et al., 2019) According to the World Health Organization (WHO), 33.5% of females and 24.1% of males in the country are obese. (World Health Organization. Saudi Arabia health profile, 2015) Obesity is linked to number of health concerns, such as diabetes, cardiovascular disease, and cancer-related morbidity and mortality, all of which lead to a reduction in both life quality and expectancy. (Alkhaldy et al., 2019, Lupoli et al., 2017) Treatment is vital for overcoming obesity’s complications. (Ruban et al., 2019) Diet, physical exercise, behavioural therapy, medications, and bariatric surgery (BS) are available treatment options. (Ruban et al., 2019, Bray et al., 20182018).

BS is considered the gold standard weight loss intervention. (Ahmed et al., 2020) Bariatric procedures can be either restrictive (gastric band, gastric balloon, and laparoscopic sleeve gastrectomy [LSG]), mainly malabsorptive (biliopancreatic diversion with or without duodenal switch [BPD/DS] duodenal-jejunal or jejunal bypass), or a combination of the two (Roux-en-Y gastric bypass (RYGB) and sleeve gastrectomy with duodenal switch). (Gudzune et al., 2013) RYGB and LSG are the two most common bariatric procedures in Saudi Arabia. (AlAli et al., 2020) Prior research has demonstrated LSG’s utility for treating morbid obesity. (Aldaqal and Sehlo, 2013, Ahmed et al., 2017) Despite the significant weight loss and reduction of comorbid conditions which follow BS, there are complications and nutritional risks associated with these procedures. (Beckman and Earthman, 2013) For instance, RYGB and LSG can potentially cause malnutrition due to their malabsorptive and restricted approach. (Mohapatra et al., 2020) Many studies have shown that micronutrient deficiencies are often diagnosed after bariatric surgery. (Gudzune et al., 2013) Protein-energy malnutrition and micronutrient deficiencies, such as cobalamin, folate, calcium, iron, transferrin, and fat-soluble vitamins are examples of malnutrition post-BS. (Hasan et al., 2020) Furthermore, BS decreases stomach acid, which in turn reduces the absorption of iron. (Smelt et al., 2020) In addition, these issues are intensified by the patient’s non-compliance in taking the required supplements. (Hasan et al., 2020) Therefore, adherence to post-surgery dietary recommendations is key to avoiding deficiencies and increasing the likelihood of successful short-term surgical outcomes. (Alkhaldy et al., 2019) These recommendations include advice on diet progression, dietary-related behaviours, the use of required supplements, nutritional therapy, and alleviating common gastrointestinal (GI) symptoms. (Hasan et al., 2020) There is insufficient data about adherence levels among the Saudi population.

Moreover, patients’ dietary intake usually reduces post-surgery due to many factors. (Al-Najim et al., 2018) Pre-meal cognitive and sensory signals that encourage food consumption, the fear of weight gain, abdominal pain, nausea, vomiting, difficulty swallowing due to texture, and plugging have all been reported as barriers to food intake after BS. (Al-Najim et al., 2018, Ziadlou et al., 2020) Through influencing meal preferences and intake, taste also directly impacts eating habits. (Al-Najim et al., 2018) Alterations in gut-derived variables, such as gut hormones, nutrition, bile acids, microbiota, and neural signals acting peripherally and centrally on homeostatic and hedonic brain areas may influence these postsurgical eating behaviour changes. (Zakeri and Batterham, 2018).

Furthermore, hormonal changes might affect dietary intake. (Zakeri and Batterham, 2018) Decreases in insulin secretion can result in a reduction of the required amino acid to produce serotonin, thereby leading to mood swings and anxiety. (Goossens et al., 2009, Jenkins et al., 2016) Anxiety has been linked to over-eating through decreased self-discipline. (Al-Najim et al., 2018) Information from the Dutch Eating Behaviour Questionnaire (DEBQ) suggests that higher levels of uncontrollable or emotional eating are linked to lower weight reduction success post-surgery. (Bryant et al., 2020) To the best of our knowledge, there are no available studies about the effects of BS on the mood and eating behaviour of the citizens of Saudi Arabia.

Therefore, this study seeks to determine the influence of BS on eating behaviour during one year of bariatric surgery, as well as the prevalence of nutrient deficiency and the level of commitment to diet and lifestyle recommendations among patients who underwent BS at King Abdullah Medical City (KAMC) in Makkah, Saudi Arabia.

## 2 Subjects and methods

### 2.1 Study design and setting

This retrospective follow-up study was performed at KAMC in Makkah, Saudi Arabia.

### 2.2 Participants

A total of 160 patients who underwent BS during 2019 at KAMC were recruited in this study. Inclusion criteria were male and female patients who aged between 18 and 65 years with a body mass index (BMI) of ≥ 35 kg/m^2^ who underwent any BS during 2019 and were followed-up for a year. We excluded pregnant women, any patients who underwent BS in the previous years, and those who had been diagnosed with psychological disorders.

The study achieved a response rate of 64%. Among the initial sample of 250 patients who underwent BS in 2019, 50 records were eliminated due to duplications, and another 40 patients were excluded due to incomplete data or complications requiring surgical intervention.

### 2.3 Data collection

Data about patients’ medical and weight history were collected from the hospital’s Trakcare® system. The data included type of BS, history of chronic diseases, blood values of haemoglobin, albumin, iron, vitamin B_12_, folic acid, vitamin D, and calcium concentrations. All patients received daily multivitamin supplement (MSV) tablets (e.g., folic acid 1 mg, calcium carbonate 600 mg, Centrum©, vitamin D_3_ 50000 IU, and iron sulfate). Data were recorded at three points of time: before the surgery, and two follow-up visits 3 and 12 months after surgery. BMI was computed by dividing weight by height squared.

The DEBQ measured the eating habits of BS patients. (Subramaniam et al., 2017) To ensure the reliability and validity of the study questions, the questionnaire was designed by a third party who had experience creating qualitative questionnaires. The questionnaire was based on previous studies. (Subramaniam et al., 2017, Hasan et al., 2020) A pilot study of 25 participants was conducted to validate the questionnaire and test the questionnaire under survey conditions, in addition to determining the duration of the interview. The questionnaire is a three-part evaluating tool that includes 13 items on the emotional eating scale, 10 items on the external eating scale, and 10 items on the restraint eating scale. The questions assessing these three separate behaviours occurred in random order and were answered using a Likert scale with the following scoring system: 1 = never, 2 = seldom, 3 = sometimes, 4 = often, and 5 = very often.

The amount of adherence to essential post-surgery food and lifestyle guidelines was examined using a questionnaire developed by Hasan and his colleagues. (Hasan et al., 2020) Participants were asked to rate how often they followed each recommendation: all the time, most of the time, some of the time, or rarely/never. Both questionnaires were completed over a phone interview.

### 2.4 Statistical analysis

Data analysis was performed using the Statistical Package for the Social Sciences (SPSS) (IBM SPSS Statistics for Windows, Version 23.0. Armonk, NY: IBM Corp). Frequency and percentages were used to display categorical variables. Data were transformed to a normal distribution. We used minimum, maximum, mean, and standard deviation to present continuous variables. We also used a paired *t*-test test to determine the presence of a significant difference in BMI and laboratory findings pre-operative (pre-op) and post-operative (post-op) at the 3- and 12-month intervals. The level of significance was set at 0.05.

## 3 Results

A total of 160 patients (56 male, and 104 female) participated in the study. The minimum age of patients was 19, the maximum was 62, and the mean was 39.73 ± 9.76 years.

Participants’ medical history was as follows: 25 (15.6%) had hypertension, 22 (13.8%) had diabetes, 5 (3.1%) had asthma, 5 (3.1%) had hypothyroidism, 2 (1.3%) had ischemic heart disease, and 3 (1.9%) had other diseases. 98 (61.2%) of the patients had no health conditions.

Table 1 demonstrates the patients’ scores in emotional, external, and restrained eating. The mean score for emotional eating was 34 ± 3.8. Around 35.6% (n = 57) of the participants had a low level of emotional eating, while 103 (64.4%) showed a moderate level. On the other hand, the external eating mean score was 20 ± 2.3. Over half (58.1%) showed a low level of external eating, while 67 (41.9%) had a moderate level. The mean score for restrained eating was 31.6 ± 2.5. Over three-quarters of the participants (77.5%) had a moderate level of restrained eating, while 36 (22.5%) had a high level.

**Table 1:** Frequency of emotional, external, and restrained eating score among adult patients after one year of BS (n = 160).

Emotional Eating Level		
Level	n	%
Low Level of Emotional Eating (Score of 32 or Less)	57	35.6
Moderate Level of Emotional Eating (Score Between 33 and 48)	103	64.4
High Level of Emotional Eating (Score of 49 and Higher)	0	0
External Eating Level		
Low Level of External Eating (Score of 20 or Less)	93	58.1
Moderate Level of External Eating (Score Between 21 and 30)	67	41.9
High Level of External Eating (Score of 31 and Higher)	0	0
Restrained Eating Level		
Low Level of Restrained Eating (Score of 22 or Less)	0	0
Moderate Level of Restrained Eating (Score Between 23 and 33)	124	77.5
High Level of Restrained Eating (Score of 34 and Higher)	36	22.5

Table 2 shows the patients’ adherence to dietary and lifestyle recommendations. The minimum was 15, the maximum was 24, and the mean was 20.13 ± 1.63. Most of the participants had a moderate adherence level, 128 (80%, or a score of between 15 and 21), and 32 (20%) had a high level (a score of 22 or higher).

**Table 2:** Frequency of participants’ adherence level to dietary and lifestyle recommendations among adult patients after one year of BS (n = 160).

Adherence to Dietary and Lifestyle Recommendations Level		
Level	n	%
Low Level of Adherence (Score of 14 or Less)	0	0
Moderate Level of Adherence (Score Between 15 and 21)	128	80
High Level of Adherence (Score of 22 and Higher)	32	20

Table 3 shows the change in BMI values pre-op and post-op at a 12-month interval. Before surgery, about 89% of the participants had class 3 obesity. 12 months after the surgery, only 3 (1.9%) achieved a BMI of normal weight, 13 (8.1%) were overweight, 35 (21.9%) were obese class 1, 47 (29.4%) were obese class 2, and 62 (38.8%) were obese class 3.

**Table 3:** Frequency of BMI changes before and after BS among adult patients (n = 160).

Pre-Op	n	%	12 Months Post-Op	n	%
BMI					
Normal Weight	–	–	Normal Weight	3	1.9
Overweight	–	–	Overweight	13	8.1
Obesity Class1	–	–	Obesity Class1	35	21.9
Obesity Class 2	3	1.9	Obesity Class 2	47	29.4
Obesity Class 3	157	98.1	Obesity Class 3	62	38.8

**Fig. 1:**
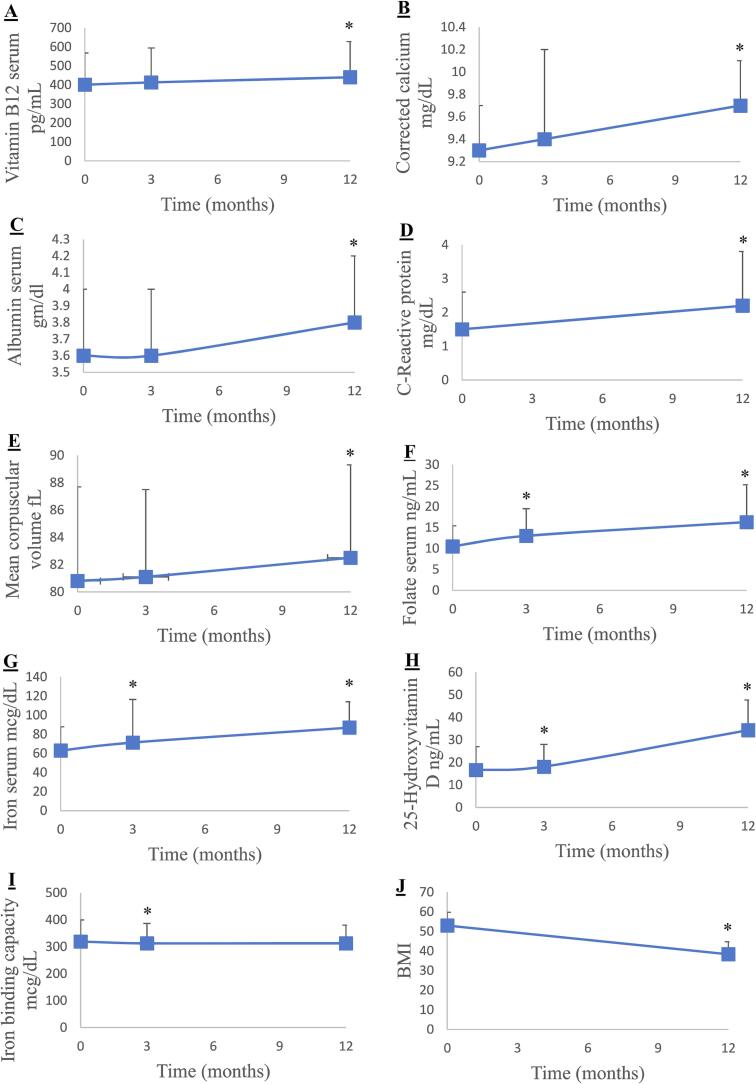
Changes in mean serum measurements Pre- and Post-Op among adult BS patients A) Blood concentrations of vitamin B_12_, B) Corrected calcium, C) Albumin, D) C-Reactive protein, E) Mean Corpuscular Volume, F) Folate, G) Iron serum, H) 25-hydroxyvitamin D, I) Iron binding capacity, and J) BMI. *Significant from the baseline measurements.

As shown in graphs B, C, D, and E of Fig. 1, when comparing pre-op blood values to post-op values 12 months later, we noted a significant increase in the mean corpuscular volume (MCV), albumin and calcium, and C-reactive protein (CRP) (all p < 0.001). As for the BMI, we observed a significant decrease at the 12-month interval (p < 0.001), as can be seen in Graph J. Graph A shows a significant increase in mean vitamin B12 (p = 0.009) at the post-op 12 month-interval.

However, as seen in Graph F, folate values significantly increased at both the 3- and 12-month post-op intervals (both p < 0.001). Serum iron (see graph G) was significantly higher at the 3- and 12-month post-op intervals (p = 0.013 and p < 0.001, respectively). Vitamin D levels (see Graph H) were significantly higher at both post-op intervals (p < 0.001). We also noted a significant decrease in the mean iron binding capacity (p = 0.05) at the 3-month post-op interval, as shown in Graph I of Fig. 1.

Table 4 shows that no significant difference was found for post-op hemoglobin, hematocrit, and red blood cells count.

**Table 4:** Comparison of mean laboratory findings during one year following BS among adult patients.

Blood tests	Value (Mean + Standard Deviation)	P-Value
Haemoglobin		
Pre-Op	12.95 ± 1.81	
3 Months Post-Op	12.85 ± 1.84	0.143
12 Months Post-Op	12.82 ± 1.78	0.236
Haematocrit		
Pre-Op	40.28 ± 5.12	
3 Months Post-Op	40.19 ± 4.96	0.744
12 Months Post-Op	40.35 ± 5.69	0.855
Red Blood Cells Count		
Pre-Op	5.28 ± 3.22	
3 Months Post-Op	4.96 ± 0.58	0.211
12 Months Post-Op	4.90 ± 0.57	0.142

A paired *t*-test was performed to compare 3- and 12-month post-op values with pre-op ones.

## 4 Discussion

This study sought to examine how BS affects eating behaviour over the course of one year, as well as to determine the prevalence of nutrient deficiency and the level of commitment to diet and lifestyle recommendations among BS patients in Makkah City.

BS is generally considered the gold standard of weight loss treatments. Weight loss following BS can be explained by such processes as decreases in food intake, absorptive area and GI secretions, and hormonal changes, as well as by surgery-related side symptoms, such as vomiting and food intolerance. (Ionut and Bergman, 2011) The data presented above reveals a significant drop in average BMI 12 months after the surgery, from 52.9 to 38.3. This is consistent with earlier findings. (Mulla et al., 2018, Kenngott et al., 2019, Khosravi-Largani et al., 2019).

Patients who undergo BS must follow dietary and lifestyle advice to achieve long-term health and weight goals. The patients in this study showed a medium-to-high level of adherence to dietitians’ instructions. Additionally, as per the advice given to them, the participants showed a firm commitment to avoiding high-calorie snacks, which will have a significant impact on their weight and overall health. This high level of adherence could be explained by the relatively short time period (i.e., 12 months) following their surgery. This has previously been observed in the literature, (Hasan et al., 2020, Lin et al., 2021) wherein patients most adhere to food and lifestyle recommendations prior to surgery and shortly afterward; however, as time passes, the rate of adherence drops. Furthermore, research has shown that, approximately 1–2 years after surgery, energy consumption, appetite ratings, and psychopathology decrease, whereas dietary adherence, supplement intake, and overall well-being tend to rise. (Bryant et al., 2020).

Increasing the inflammatory process is linked to obesity and heavily influenced by adipose tissue. (Radi et al., 2017) Weight loss leads to an enhanced inflammatory markers profile. Indeed, a Canadian study demonstrated that at 12 months post-LSG, both body weight and CRP significantly decreased. (Randell et al., 2018) However, our results showed that serum CRP significantly increased in the 12 months following the surgery. This could be explained by the influence of such external variables as high-fat and high-calorie diets, illnesses, mental stress, and oxidative stress – all of which are thought to stimulate the immune system. (Radi et al., 2017) Several studies in humans have found a substantial relationship between high-sensitivity C-reactive protein (hsCRP) and IL-6 and various dietary variables, implying that inflammatory biomarker concentrations could be influenced by diet. (Smidowicz and Regula, 2015, Ko et al., 2014) Moreover, food can change the composition and characteristics of gut microbiota, and the balance of pro- and anti-inflammatory gut microbiota populations, which can affect inflammation. (Kim et al., 2019, Tomova et al., 2019, Wang et al., 2021, Bolte et al., 2021) Our participants may consume less fruit and vegetables because of a reduction in their general dietary intake due to the effects of BS on GI tract.

We observed a high level of serum albumin in the patients 12 months after their BS. Serum albumin concentration is a helpful tool for determining the nutritional status of proteins. (Lupoli et al., 2017) This means patients were consuming enough protein to guarantee their positive impact on body composition and prevent malnutrition. (Steenackers et al., 2018, Hosseini-Esfahani et al., 2021) Furthermore, the tolerance for protein-rich diets tends to improve after one year of performing the surgery. (Isom et al., 2014) Moreover, when comparing minerals levels in the blood at the pre- and 12-month post-op stages, we noted a considerable rise in calcium. Our results accord with Antoniewicz and his colleagues, who found that calcium concentration increased significantly within the LSG group and during the 1- and 12-month follow-ups. (Antoniewicz et al., 2019) This might be as a result of enhanced bone remodelling following BS, in which calcium levels may change. (Antoniewicz et al., 2019) This increase among our participants could be explained by their receiving daily 600 mg calcium carbonate tablets, as well as the fact that the majority took the recommended vitamin and mineral supplements with a modest-to-high level of adherence. In addition, our findings show that, when compared to pre-op levels, vitamin D levels were considerably higher at the 3- and 12-month post-op intervals. It has been found that vitamin D deficiency and insufficiency were common pre-surgery, ranging from 33.1 (23.9) nmol/L at baseline to 57.1 (23.1) nmol/L at 12 months post-surgery after receiving routine supplementation. (Fox et al., 2020) To prevent vitamin D deficiency following SG and RYGB, it has been suggested that patients should consume vitamin D between 2000 and 4000 IU of vitamin D_3_ per day. (O'Kane et al., 2020) All of the patients in our research who experienced vitamin D deficiency and insufficiency were given vitamin D_3_ 50000 IU nutritional supplements only on a weekly basis for 8 weeks.

As the B complex vitamins are water-soluble, they cannot be stored in substantial quantities in the body, thus necessitating a constant supply. (Moore and Sherman, 2015) Consequently, MVSs are commonly provided so as to prevent vitamin deficits. (Smelt et al., 2020) Regarding vitamin B_12_ levels 12 months after surgery, we noted an increase in the mean. Deficiencies in vitamin B are more common after malabsorptive treatments, such as RYGB, but less common after such highly restrictive procedures as SG. (Lewis et al., 2020) This could explain our result, since the majority of our participants underwent SG. Additionally, our patients showed proper nutritional status because of their adherence to a multivitamin/multimineral program and their taking of supplements with adequate levels of vitamins and/or minerals. Our participants took Centrum®, which contains 1 mcg of B_12_.

Within our data, postoperative serum folate levels rose significantly, which is similar to earlier findings. (Moore and Sherman, 2015) Our patients consumed supplements with suitable levels of 1 mg of folic acid. It is well known that micronutrient supplementation is recommended for all post-operative patients, according to evidence-based guidelines, so as to prevent any deficiencies. (Parrott et al., 2017, Mechanick et al., 2019).

Iron deficiency can occur after GS due to the reduction in absorption, meaning that supplements are essential for preventing deficiencies. (Ruz et al., 2012, Hany Emile and Elfeki, 2017) However, we did not observe any iron deficiencies, but instead noted that serum iron rose from 63 to 71.4 at 3 months and to 86.9 at 12 months and iron binding capacity (IBC) fell from 318.8 to 312.4 at the 3-month interval. To avoid post-surgery iron deficiency, our patients were given oral iron in the form of ferrous sulfate (190 mg every 12 h). Lefebvre et al. (Lefebvre et al., 2021) found that transferrin IBC was 62.7 at the baseline, and reduced to 61.8 at 12 months. Additionally, a previous study resulted that the percentage insufficiency of serum iron was 29.8% at the start of their study, but dropped to 15.6% after one year., Another finding of our research was the substantial rise in the mean MCV detected when pre-op blood values were compared to 12-month post-op values. In a recent study, Lefebvre et al. (Lefebvre et al., 2021) found that MCV was 85.2 at the baseline and 86.6 at the end of the 12-months.

Regarding eating behaviours, we observed that approximately 64.4% of research participants had a moderate level of emotional eating, with the remainder having a low level. Over half (58.1%) of the participants had a low level of external eating, while 41.9% exhibited this behaviour to a moderate degree. It has been reported that anxiety, depression, and disappointment are among the factors that influence both emotional (Bilici et al., 2020) and external eating (Sevinçer et al., 2017). When asked whether feeling of depression or discouragement were accompanied by a desire to eat, 45% of cases, answered ‘sometimes’. Furthermore, higher DEBQ scores for external and emotional eating have been linked to decreased weight reduction success. (Pepino et al., 2014) A person’s psychological eating behaviour can influence their capacity to adjust to post-surgery dietary modifications, which can either hamper or facilitate weight reduction success. (Bryant et al., 2020) The regulation of post-surgery eating behaviour is both complicated and impacted by a variety of factors. (Emilien and Hollis, 2017) Sex, type-2 diabetes, genetics, and the type of bariatric procedure all influence changes in eating behaviour after surgery. (Zakeri and Batterham, 2018) Furthermore, eating behaviour changes post-BS are thought to be mediated through gut–brain signalling pathways. (Zakeri and Batterham, 2018).

There are various strengths and limitations to the current study. To the best of our knowledge, this is the first study in Saudi Arabia to look into BS and eating habits, as well as vitamin deficiency, and adherence to dietary and lifestyle recommendations. We used a validated questionnaire to assess eating behaviour. We opted to use serum transferrin receptors or total IBC due to their being more accurate indicators of iron deficiency than serum iron or ferritin, which can be raised in the inflammatory state of obesity. (Gletsu-Miller and Wright, 2013) Moreover, the fact that the participants were recruited from one of Saudi Arabia's leading government obesity treatment centers can be regarded as a strength. The study's retrospective approach posed a constraint in terms of limits as we could not control the exposure or outcome assessment, and instead were forced to rely on others for accurate recordkeeping. The measurement of serum biochemical parameters prior to and during the surgery was not supported by sufficient information. Additionally, there are no data on preoperative food intake. The study was further limited by its small sample size. The diet and physical activity history of the participants was not assessed in the study, nor did we assess their eating behaviours pre-surgery and three months post-surgery. For a fuller analysis, a multicentre study with a larger sample size and various sociodemographic features could be conducted. Future research should examine the link between BS and CRP levels, as well as the connection between BS and dietary habits before and after surgery. More research is needed to determine the severity of anemia. Psychological factors should also be investigated further so as to comprehensively understand the possible mechanisms which might influence weight loss and the maintaining of an ideal weight after surgery. Studying weight loss drugs as a potential alternative to bariatric surgery is notable, particularly in terms of how they affect eating behaviour, the prevalence of nutrient deficiencies, and the level of adherence to diet and lifestyle recommendations. Supplements may attract heightened attention due to their observed adverse effects, and one such supplement that may exhibit a positive correlation with the occurrence of ischemic heart disease is calcium.

## 5 Conclusions

In conclusion, the results at the 12-month assessment of BS reveal remarkable improvements in the plasma levels of various nutrients, including vitamin B_12_, folate, vitamin D, iron, corrected calcium, albumin, CRP, and MCV, indicating positive responses to the intervention. Furthermore, a significant decrease in BMI is observed, demonstrating the effectiveness of the dietary and lifestyle recommendations. The moderate to high adherence to these recommendations further suggests the feasibility of implementing long-term changes. Notably, the prevalence of emotional and restrained eating behaviors is moderate, showing potential areas for targeted interventions. However, the relatively low incidence of external eating is encouraging, indicating a positive step towards healthier eating habits. Overall, these findings underscore the positive impact of the intervention on nutritional status and highlight the importance of continued efforts to promote balanced dietary habits and encourage a healthy lifestyle.

## Declaration of Competing Interest

The authors declare that they have no known competing financial interests or personal relationships that could have appeared to influence the work reported in this paper.

## Data Availability

Data will be made available on request.
